# Recent advances in functional region prediction by using structural and evolutionary information – Remaining problems and future extensions

**DOI:** 10.5936/csbj.201308007

**Published:** 2013-12-05

**Authors:** Wataru Nemoto, Akira Saito, Hayato Oikawa

**Affiliations:** a Division of Life Science and Engineering, School of Science and Engineering, Tokyo Denki University (TDU), Ishizaka, Hatoyama-cho, Hiki-gun, Saitama, 350-0394, Japan

**Keywords:** bioinformatics, structure, sequence, protein design

## Abstract

Structural genomics projects have solved many new structures with unknown functions. One strategy to investigate the function of a structure is to computationally find the functionally important residues or regions on it. Therefore, the development of functional region prediction methods has become an important research subject. An effective approach is to use a method employing structural and evolutionary information, such as the evolutionary trace (ET) method. ET ranks the residues of a protein structure by calculating the scores for relative evolutionary importance, and locates functionally important sites by identifying spatial clusters of highly ranked residues. After ET was developed, numerous ET-like methods were subsequently reported, and many of them are in practical use, although they require certain conditions. In this mini review, we first introduce the remaining problems and the recent improvements in the methods using structural and evolutionary information. We then summarize the recent developments of the methods. Finally, we conclude by describing possible extensions of the evolution- and structure-based methods.

## Introduction

**Figure 1:**
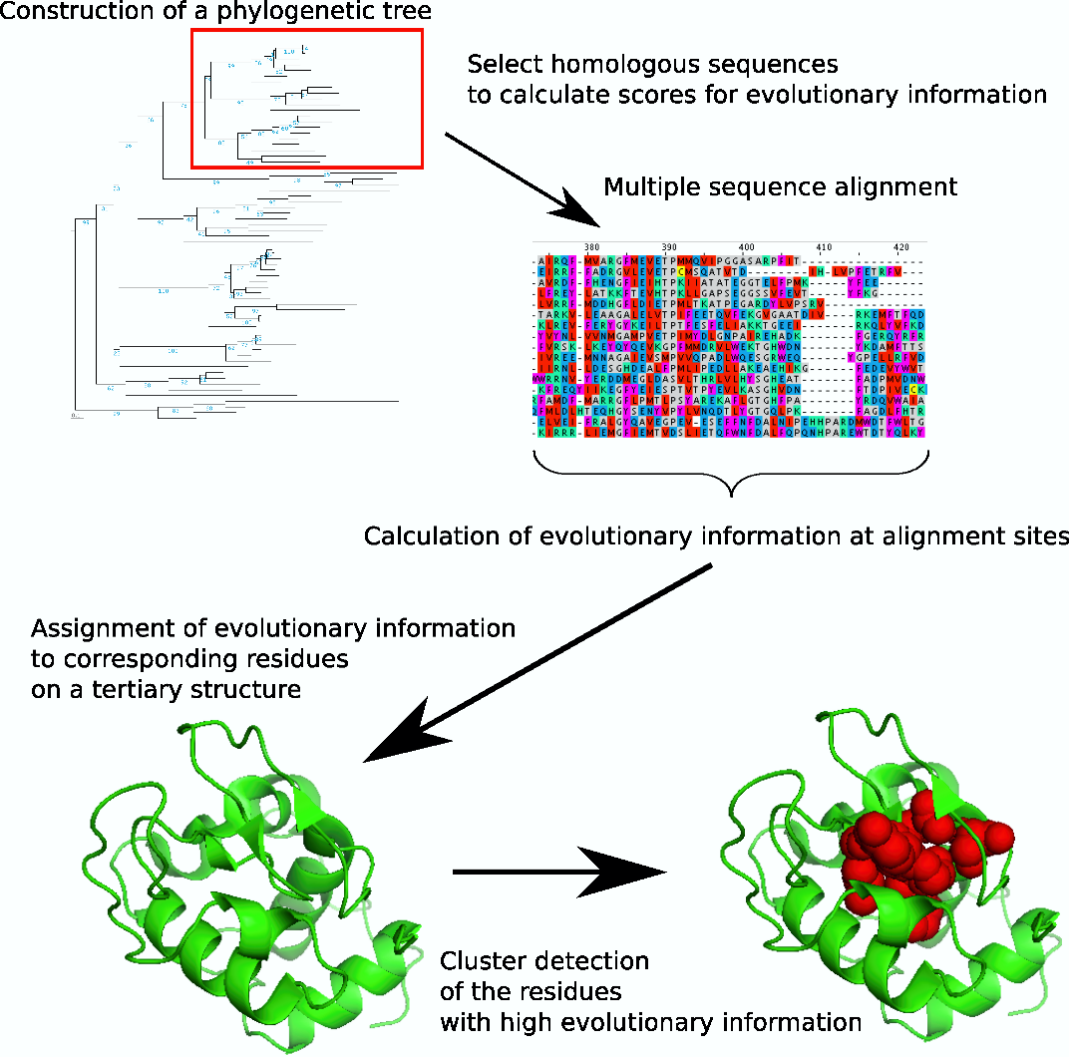
Procedure of the methods by integrating the structural and evolutionary information.

The prediction of functional regions in a protein is an important research focus, and many methods have been developed for this purpose [1]. One of the most effective strategies is the detection of evolutionarily important residues on the tertiary structure of a protein, by integrating the structural and evolutionary information encoded in a multiple sequence alignment (MSA) [2–9] (see a schematic image of the strategy in Figure 1). The most popular and pioneering method based on the strategy is Evolutionary Trace (ET) [2], which uses a phylogenetic tree to rank the residues in a protein by their evolutionary importance and maps them on a closely related structure. The highly ranked residues are often clustered in space, and thus these clusters correspond to functionally important residues and are used to identify them. Many servers perform ET [10–12] or similar methods [3, 5, 7, 13], and were developed by the original designers of ET [10] or other groups [3, 5, 7, 11–13]. In this mini review, we will summarize the recent advances in the ET and ET-related methods (evolution and structure information-based methods) using structural and evolutionary information, including our work, over the past few years, and then discuss the remaining problems. First, we will summarize the various improvements of the measurements to evaluate the evolutionary information calculated from an MSA. We will subsequently introduce several improvements of functional region prediction by exploiting the structural information. We will finally introduce an important problem shared by the MSA-based methods in structural bioinformatics, and the challenges to solve it. At the end of this review, we will explain the potential extensions of the structure- and evolution-based methods. The web servers of the introduced methods and their update statuses are summarized in Table 1.

**Table 1:** The computational methods to predict functional regions by using evolutionary and structural information, discussed in this review, which are available through the internet. Names (abbreviation) and classification of each service (Server or Database), URLs, and their features are described in each column.

Name (Server/Database)	URL	Description
**ET (Server)**	http://mammoth.bcm.tmc.edu/ETserver.html	**Pioneering work** Last updated: 2011
**Crescendo (Server)**	http://mordred.bioc.cam.ac.uk/∼crescendo/crescendo.php	**Discrimination between structural and functional constraints** Structural and functional constraints are discriminated by the application of ESST. Last updated: 2006
**CUBE-DB (Database)**	http://epsf.bmad.bii.a-star.edu.sg/cube/db/html/home.html	**Integration of conservation & other scores.** Pre-evaluated conservation and specialization scores for residues in paralogous proteins are provided as a table. Last updated: 2012
**JET (Server)**	http://www.lgm.upmc.fr/JET/JET.html	**Integration of conservation & other scores** Integrated scores of residue conservation and physicochemical properties are used for the prediction. Last updated: 2009
**SitesIdentify (Server)**	http://www.manchester.ac.uk/bioinformatics/sitesidentify/	**Conservation & other scores** Conservation scores and geometry-based cleft identification are used for the prediction. This server may no longer be updated.
**Direct Coupling Analysis (DCA)**	http://dca.upmc.fr/DCA/DCA.html	**Coevolutionary relationship between two sites** Discrimination between directly and indirectly correlated residues was achieved by direct-coupling analysis (DCA). Last updated: 2012
**MISTIC (Server)**	http://mistic.leloir.org.ar/index.php	**Visualization of coevolving sites** Connectivity among coevolving sites is visualized by a circular representation of the MI network. Last updated: 2013
**ETA (Server)**	http://mammoth.bcm.tmc.edu/eta/	**Toward function prediction** A 3D template composed of ET residues is used for function prediction. Last updated: 2013
**FREPS (Server)**	http://freps.cbrc.jp	**Appropriate sequence selection** The MSA with the maximum DSPAC is adopted for the prediction. Last updated: 2012
**FunShift**	http://funshift.sbc.su.se	**Functional shift analysis** FunShift performs functional shift (divergence) analysis between the subfamilies of a protein domain family. The present release uses Protein Domain families in Pfam (Version 12.0). Last updated: 2004
**EvoDesign (Server)**	http://zhanglab.ccmb.med.umich.edu/EvoDesign/	**Protein design** Designing ideal protein sequences for given scaffolds by evaluating the foldability and goodness of the designs. Last updated: 2013

## Recent advances

### Improvements in the methods to evaluate evolutionary information

One of the most widely used scores to consider evolutionary information is the residue conservation at a site in an MSA. The residue conservation reflects the evolutionary selection at functional sites to maintain protein function and to retain structural folds [6], regardless of the developed conservation score formulae [14]. Therefore, the discrimination between the functionally important residues and the structurally important ones is often difficult [6]. This problem has led to limitations of the methods to predict the functional regions using conservation scores. In order to distinguish between the residues conserved for functional reasons and those conserved for structural constraints, Chelliah *et al*. [6] developed Crescendo. This program calculates the conservation scores with an Environment-Specific Substitution Table (ESST) [15], which describes the patterns of substitutions in terms of the amino acid locations within secondary structure elements, as well as the solvent accessibility and the existence of hydrogen bonds between side chains and neighboring residues. Crescendo [6] predicts functional regions by identifying clusters of residues with unusually high evolutionary restraints. To this end, they identified the evolutionary restraint at a site, as follows: 1) whether there is a high degree of evolutionary conservation than expected, 2) whether ESST makes weak predictions of the substitution patterns, and 3) whether there are residues within spatially conserved regions, when protein structures within the same family are superimposed. Cheng *et al*. [16] also addressed a similar problem, and developed a method to predict the functional regions by distinguishing between functional constraints and structural constraints, but they adopted a different strategy to estimate the structural constraint. In order to obtain measurements of the structural constraints in a protein structure, they used Rosetta [17], which is a computational method to design a protein and calculate its free energy. They showed that combining these measures with sequence conservation improved the prediction of functional protein sites.

Zhang *et al*. [18] developed CUBE-DB, which provides calculated conservation and specialization scores for residues in paralogous proteins. The advantage of their database is that the functional specificity at a site is calculated by considering two models of evolution after divergence, “heterotachy” and “homotachy”. The word heterotachy (for “different speed” in Greek) was applied by Lopez *et al*. [19] to refer to within-site rate variations throughout time in the field of molecular evolution. In contrast, homotachy (for “same speed” in Greek) refers to the state in which the evolutionary rate of a position is constant throughout time. Heterotachy was found among homologous sequences of distantly related organisms, often with different functions. In such cases, the functional constraints are likely to be distinct, which would explain the different distributions of variable sites. Zhang *et al*. [18] used heterotachy for referring to the evolutionary rate variations among homologous groups. A high score is calculated at a site where the residues are conserved in the reference group of orthologs, but they overlap poorly with the residue type choices in the paralogous groups (such positions are referred to as functional determinants). In contrast to the case of heterotachy, homotachy requires the conservation at a site within each paralogous group (referred to as functional discriminants). Residues with high scores are mapped on an evolutionarily related structure, if available, via Jmol [20], *etc*., and are summarized as a table (html or downloadable xlsx format). CUBE-DB presently covers only human proteins belonging to multi-member families.

### Integration of conservation and other scores

It is difficult to predict all of the types of functional regions by a single score, because each functional region has its own physico-chemical properties. For example, protein interfaces are not simply discriminated from non-interface surfaces by the patches of conserved residues [21]. The most conserved patches of residues overlap in only 37.5% of the actual protein interface, although the properties of the interface differ from those of the rest of the protein. Considering other types of scores seems to be essential, to improve the prediction accuracy. Engelen *et al*. [22] addressed this problem by integrating the conservation information and the specific physicochemical properties of the residues. They developed the Joint Evolutionary Trees (JET) method [22], to detect protein interfaces, the core residues involved in the folding process, and the residues susceptible to site-directed mutagenesis and relevant to molecular recognition. The performance of JET is better than those of the other state-of-the-art methods.

Teppa *et al*. [23] compared the abilities of several methods (real-valued ET [24], cumulative mutual information (MI) [25], proximity MI [25], evolutionary trace integer value [2], and the methods designed for the identification of SDPs (SDPfox and XDET) [26]) to identify catalytic residues in enzymes, in order to investigate the extents to which the predictive powers of the different methods overlap. The results revealed that the methods can be divided in three groups, with limited mutual overlaps. These groups consist of the methods in which the predictive signal is strongly correlated to the sequence conservation, those in which the predictive signal is derived from MI, and those developed for the prediction of specificity-determining positions. Interestingly, the combined scores of the first and second groups (sequence conservation group and MI group) achieved the highest performance. These observations revealed that the sequence conservation and the MI scores are considered to be distinct signals encoded on the MSA, and produce a complementary effect. Therefore, their results demonstrated the possibility of detecting catalytic residues more accurately, by integrating structural and higher-order sequence evolutionary information. Thus, the integration of the conservation score with other types of scores represents a trend toward improving evolutionary information methods. In addition to the methods examined in Teppa's work [2, 24–26], Bray *et al*. [27] developed a functional site prediction tool, SitesIdentify, which is based on combining sequence conservation information with geometry-based cleft identification. This method functioned quite favorably in comparison to other methods, in the active site predictions for 237 non-redundant enzymes. As of 1^st^ November, 2013, the SitesIdentify server is not working at the URL described in the original paper [27].

### Coevolutionary relationship between two sites

The evolutionary scores calculated in an MSA used for functional region prediction are roughly divided into two types: the scores at a site and those between sites. The former type is the conservation/variation of amino acids at a site, while the latter one is the score of the degree of coevolutionary relationship between two or more sites. In our opinion, the performance comparisons by Teppa *et al*. [23], described in the previous section, only focused on the former point: the conservation/variation of amino acids at a site. Several methods, such as correlated mutation [28, 29], MI [30–36], and covariation [37], have been developed to estimate the degree of the coevolutionary relationship between two sites among the sequences in an MSA. The methods in the former group are based on conserved residues in MSAs, but the methods in the latter group that detect coevolution between two sites are based on variable sites. Furthermore, it should be noted that invariable sites do not contain any information in the coevolutionary score. A high coevolutionary score is considered to indicate the spatial proximity or functional connectivity between the sites, even when the sites are not close in the primary structure of a protein. Therefore, these methods have been applied to detect not only intramolecular interactions [28–38] but also intermolecular interactions [39], regardless of direct or indirect interactions. Aguilar *et al*. [40] investigated how coevolution information can be used to improve the prediction methods for functional residues. They found that the clusters of co-evolving sites related to the catalytic sites of an enzyme have distinguishable topological properties in the residue-residue interaction network, and also observed that these clusters usually evolve independently. Interestingly, they suggested that the clustering of coevolving residues could be related to a fail-safe mechanism, which causes no harm or minimizes harm to other parts in a protein structure, in the case of a functional loss at a site. Kowarsch *et al*. [41] performed comprehensive analyses of point mutations causing human diseases, with respect to the correlated mutations. They showed that 1) the correlated sites are significantly more likely to be disease-associated than expected, 2) these signals cannot be explained by the conservation patterns at each site, and 3) many correlations are not related to physical contacts between sites. However, Halperin *et al*. [42] highlighted the limitation of correlated mutation analyses, which might also be true for other coevolutionary relationship-based approaches. They showed that several correlated mutation methods achieve practical accuracy for intramolecular interaction prediction on their dataset, but the accuracy declines for intermolecular interaction prediction. Overall, they insisted that the examined methods are not suitable for large-scale intermolecular contact predictions. In other words, the current methods can only achieve practical accuracy for a handful of families. Therefore, the potential for the application of coevolutionary information to functional region prediction remains debatable. In addition, these methods have an important shortcoming that is considered to affect their predictive accuracies. One important problem stems from the fact that correlation in amino acid substitution may arise from direct as well as indirect interactions [43].

The availability of many protein sequences enables the use of various statistical approaches to address this problem. Recently, discrimination between directly and indirectly correlated residues was achieved by the direct-coupling analysis (DCA) by Weigt *et al*. [43]. DCA combines covariance analysis with global inference analysis, adopted from use in statistical physics. A message-passing algorithm was used to implement DCA (mpDCA), but it was rather costly computationally because it is based on a slowly converging iterative scheme. Hence, the same group applied an algorithm based on the mean-field approximation of DCA (mfDCA), which is 103 to 104 times faster than mpDCA, and thus can be used to analyze many long protein sequences rapidly [44]. In addition to the DCA-related methods, several groups addressed the discrimination between direct and indirect correlations [45–50]. These methods were primarily applied to identify constraints to fold a protein tertiary structure [44–49]. However, Weigt *et al*. [43] applied their method to identify constraints to maintain a protein-protein interaction [43, 51].

In addition, one of the recent advances in coevolution-based approaches is the development of MISTIC [52], by which a user can visualize the connectivity among coevolving sites as a circular representation of an MI network and their MIs interactively. Even when the initial result is too complicated to understand, other scores (cumulative MI [25], conservation, proximity MI [25] *etc*.) can be considered simultaneously at both the nodes and edges, to highlight the information encoded within an MSA. Such a visualization tool clarifies the intricate evolutionary connections among sites.

### Toward function prediction

Almost all annotations assigned to protein sequences rely primarily on the computational identification of similarity between the protein sequences with unknown and known functions, which are identified by BLAST [53] and other programs. Annotations are often misleading when the sequence similarities between the queries and the retrieved sequences are low. In these cases, the global structural similarity between a protein of unknown function and one with a known function is utilized [54–62]. Even when this approach fails, further functional information might be obtained by using local structural similarities [63, 64]. For example, surface patches or clefts [65–73], or tertiary templates of small numbers of functional residues [65, 74–81] are used as 3D templates to infer protein functions, through the identification of the corresponding key functional residues and their geometries on other structures. Such 3D templates may identify functional analogs without detectable homology that convergently perform the same function. Various characteristics of local structures have been used as queries to identify local structural similarity in distantly related- or non-homologous structures. These functional region predictions by local structural matches are often complementary to methods by global structural or sequence-based matches, when global structural or sequence-based methods do not provide detailed information about a protein of unknown function. In our opinion, however, evolutionary information has not been fully exploited for the detection of functional similarity among non-homologous structures.

Recently, Kristensen *et al*. [82] applied evolutionary information to develop the Evolutionary Trace Annotation (ETA) pipeline, which in principle can be applied to detect functional similarity among non-homologous structures. The basic idea of the ETA-based function annotation [82] is described as follows. A schematic image of the method is available at http://mammoth.bcm.tmc.edu/eta/manual.

html. At first, a few key functional residues are clustered into 3D templates, and local structures similar to them are searched for in other protein structures. Secondly, when the 3D template is matched in the structure of a protein, the function of the structure in the found protein is transferred to the query structure. In order to increase the sensitivity of the functional annotation [83], the proteins identified by ETA are linked together into a network of ETA similarities; then, starting from proteins with known functions, competing functional labels diffuse link-by-link over the entire network. A likelihood *z*-score for every function is assigned to every node. The function corresponding to the most significant score is adopted at each node, as its annotation, for example, by referring to the Enzyme Commission (EC) numbers of the retrieved structures. In high throughput controls, this competitive diffusion process recovered enzyme activity annotations with 99% and 97% accuracies at half-coverage for the third and fourth levels of the EC number, respectively, although currently these predictions have only been evaluated for homologs. These accuracies corresponded to false positive rates 4-fold lower than that of the nearest-neighbor method and 5-fold lower than that of the sequence-based annotations.

### Automatic sequence selection to evaluate evolutionary information

The selection of homologous sequences is a critical step in the prediction of functional regions by using the conservation score, because conserved residues are identified through comparisons of homologous sequences [84]. The same is true for the other evolutionary information-based methods described above. We empirically know that a certain degree of sequence divergence in the set of homologous sequences is essential for the identification of conserved residues. However, the selection of an appropriate homologous sequence to calculate residue conservation has not been sufficiently addressed. Aloy *et al*. [8] developed an automatic method to predict the functional regions of a protein, by detecting conserved residue clusters on the tertiary structure. If no cluster is identified, then the MSA is reconstructed by removing the distant homologues to the prediction target, according to the evolutionary relationships suggested by a phylogenetic tree. The process is iterated until at least one cluster is identified. In other words, the iteration process is forcibly terminated, even if more appropriate conditions are present within the untested sequence space. Mihalek *et al*. [85, 86] applied a residue clustering measure, which was originally developed as a formula for the identification of conserved residue clusters, to indicate the appropriateness of a set of sequences for functional region predictions [85, 86]. The measure quantifies the degree of clustering of the evolutionarily important residues in the tertiary structure of a protein, and attaches greater importance to the clustering of the residues that are far from each other on the primary structure. The sequence set selected by their measure performed better in their functional region prediction by the real valued ET-based method [87]. They showed that the performance of their method for protein-protein interaction interfaces was lower than that for active sites. Recently, we addressed a similar problem by a different approach, and developed the Functional REgion Prediction by using Spatial statistics (FREPS) method [84]. FREPS implements an index, DSPAC (the Degree of Spatial AutoCorrelation), to measure the appropriateness of a set of homologous sequences [84]. Structure and sequence information are integrated by spatial statistics within the index, which represents the degree of conserved residue clustering on the tertiary structure of the protein. The functional region prediction performance, using the set of sequences selected by DSPAC, was better than that obtained using the set selected under the fixed percent sequence identity-conditions. In addition, DSPAC successfully distinguished the sequence set including only C-type lysozyme from that including both C-type lysozyme and its non-enzyme homologue, α-lactalbumin. Similar to the residue clustering measure [85, 86], however, the performance of DSPAC for protein-protein interaction interfaces was lower than that for active sites, although the details have not been published yet. In order to assess various types of functional regions, DSPAC [84] and residue clustering measures [85, 86] should be improved.

## Future directions

We would like to conclude by describing three possible extensions of the methods using structure and evolutionary information. The first is the extension to identify the functional differences of closely related proteins. In considerable numbers of protein families, a subfamily develops a new function, changes substrate specificity, or loses an original function. These family members can be categorized into several subfamilies, for investigations of their functions. Several databases and methods have been developed. For example, the FunShift database [88] is a collection of such functionally changed subfamilies (function shift) in a Pfam [89] protein family, identified by using Conservation Shifting Sites and Rate Shifting Sites. It is useful for protein design and mutagenesis studies, although FunShift has not been updated since 2004. PANTHER [90] also provides similar information curated by experts. Recently, Lee *et al*. developed GeMMA [91], which automatically classifies families and superfamilies into functional subfamilies, and is comparable to the established method SCI-PHY [92]. The common feature of these methods and databases is that they do not consider structural information, which might limit their predictive accuracies. As described above and in our previous work [84], DSPAC was able to distinguish the sequence set including only the proteins with identical functional structures, from that including both the proteins with identical functions and those with different functions. Theoretically, the same is expected to be true for the residue clustering measure [85, 86]. In addition, the ETA-based search [82] could be extended to identify the functional differences by considering the local structural differences between a 3D template and a matched one. These measures might improve the predictive accuracy to identify the functional differences of closely related proteins.

The second point is the extension to the template selection in homology modeling, by the application of evolutionary information. See details in our previous work [84]. Homology modeling is frequently utilized for a sequence without a solved crystal structure. Thus, the main purpose of the modeling is to investigate the molecular function. It would be better to use the structure of a protein with a function considered to be the same as or similar to the sequence under consideration as a template, although a model based even on a template with different functions can provide important information. However, the template structures retrieved by fold recognition programs do not always have the identical or similar function to those of the target sequence, because such programs do not directly evaluate the functional similarity between the target and a retrieved structure. Here, we consider the inverse problem. Suppose that we have a structure. The problem is to determine which homologous sequences can be modeled, using the given structure as the template. Fold recognition programs may not provide an answer to the problem, since the functional similarity to the structure-known protein is not considered in these programs. Considering the functional differences by an ETA-based strategy [82], residue clustering measures [85, 86] or DSPAC [84] can be used to solve the problem, since these can be extended to identify the set of sequences that shares the same or similar biochemical functions.

The third point is the extension to protein design [93], which is becoming a popular research subject. One of the main purposes of protein design is to optimize the physicochemical characteristics of a designed structure. Most current protein design methods rely on physics-based force fields to search for low free-energy states. Recently, Mitra *et al*. [94] developed a method, EvoDesign, to design ideal protein sequences for given scaffolds. At first, EvoDesign collects a set of proteins with similar folds from the PDB data, by a structural alignment with a query structure. Then, an evolutionary profile is constructed from the MSA of the retrieved structures. This profile is used for a conformational search in sequence space, where the physicochemical packing of the side-chain and backbone atoms is accommodated by neural-network-based solvation, torsion angle and secondary structure predictions. However, this step does not consider the functional similarity with the scaffold, but mainly focuses on the foldability and goodness of the designs. Therefore, the retrieved structures might not always have the identical or similar function to that of the original scaffold. Evaluating the functional similarity by evolutionary information-based approaches would contribute to progress in protein design.
